# Nutrition Support Practices for Infants Born <750 Grams or <25 Weeks Gestation: A Call for More Research

**DOI:** 10.3390/ijerph191710957

**Published:** 2022-09-02

**Authors:** Melissa Thoene, Ann Anderson-Berry

**Affiliations:** Department of Pediatrics, University of Nebraska Medical Center, 981205 Nebraska Medical Center, Omaha, NE 68198, USA

## 1. Introduction

With advances in medical care and efforts to care for continually smaller and younger preterm infants, the gestational age of viability has decreased, including as young as 21 or 22 weeks of gestation [1]. While many extremely preterm infants (born <28 weeks gestation) [2] may be considered extremely low birth weight (ELBW, <1000 g), those born at the youngest ages often fall into the subcategory of “micropremie” (<750 g). Regarding the care of micropremies or those born in the earliest half of the extremely preterm age category (<25 weeks gestation), clinical care teams must consider the optimal approaches to managing nutritional support. Nutrition support and growth are critical components in the care of these vulnerable infants [3], so nutrition management practices must focus on two factors: (1) the provision of adequate nutrition to support “normal” growth and development, and (2) the prevention of nutrition-related complications.

Though extremely preterm or ELBW infants are at a high risk for nutrition-related complications, comprehensive evidence-based nutritional interventions for this population have not yet been established and endorsed by national working groups. Thus, evidence-based nutrition research within a micropremie patient population grows ever more deficient as younger infants are cared for in the neonatal intensive care unit (NICU) setting. Consequently, clinical care teams aiming to develop nutrition support protocols for this extreme subcategory must consider if nutrition practices for a broader population of extremely preterm or ELBW infants are fully applicable at the smallest weights and periviable gestational ages. As recognized within the World Review of Nutrition and Dietetics *Nutritional Care for Preterm Infants*, “the optimal nutrition of the critically ill VLBW (very low birth weight, <1500 g) infant is largely unknown” [4], highlighting a deeper lack in the smallest infants. It is recognized that “consensus” or “expert” opinions may exist regarding the optimal strategies for nutritional management in preterm infants [4]. While guiding in the presence of conflicting or limited data, these opinions may not be solely evidence-based nor adequately compare the immediate or life-long physical or developmental effects resulting from specific nutritional interventions. Thus, more evidence-based research is necessary to determine the optimal nutritional interventions for the population of infants born micropremie (<750 g) or at gestational ages <25 weeks.

## 2. Physiologic Considerations

*Why do we consider this subcategory of preterm infants unique?* Wide proportional differences exist among a categorized population of extremely preterm and ELBW infants. For an example from the 2013 Fenton preterm infant growth chart, the average size (z-score = 0) ranges from ~480 g for a female at 22 0/7 weeks to ~950 g for a male at 27 0/7 weeks [5]. While both instances are generalized as extremely preterm and ELBW, this spans a 2× increase in body size and a more than 20% longer duration of intrauterine growth and development before birth. Thus, we must consider if these relatively large proportional differences indicate the need for more tailored enteral and parenteral nutrition management strategies within the subcategories of the extremely preterm and ELBW population.

These considerations are stressed when reviewing data on fetal lung development. Many extremely preterm infants will be in or transitioning to the saccular stage of lung development; however, infants born <24 weeks will still be in the canalicular stage, with little to no production of surfactant [6]. Additional focused research may allow us to identify whether macronutrient- or micronutrient-related needs are altered during these earliest stages of lung development. Furthermore, it may be theorized that the lung tissue during the canalicular phase is fragile and that inflammatory exposures (pre or postnatally) and alterations to its physical structure caused by life-saving medical interventions may alter the normal trajectory of development, therefore contributing to a later development of bronchopulmonary dysplasia. Hence, future research may help determine whether lung tissue integrity at these earliest developmental stages can be preserved or whether inflammatory damage can be lessened by specific early-life nutritional interventions, such as the supplementation of Vitamin A [7] or antioxidative micronutrients [8].

How might the developmental stages of other organs—such as the intestinal tract— impact nutrition support strategies in this subset of preterm infants? Future research will guide us more concretely on these answers, but the growth of other organs besides the lungs is rapid and predisposition in early life to their proper or impaired functioning will lead to healthy or diseased states in later life. A review of the prolific growth and organogenesis in fetuses is truly astonishing and must be considered in the context of nutritional research and clinical care in an extreme subpopulation. For example, (1) the fetal brain is estimated to grow at a rate of 250,000 nerve cells per minute to achieve more than 100 billion nerve cells by the time of normal term birth [9], and (2) the gastrointestinal tract contains hundreds of millions of neurons [10] and grows from approximately 125 to 200 to 275 cm from 20 weeks to 30 weeks and finally to term gestational age [11]. Consequently, an inappropriate delivery of nutrition during these critical periods impairs the potential for proper or complete organ development and function. For example, an insufficient level of vitamin A during fetal development may be associated with a smaller kidney size [12,13] or a lower number of accrued nephrons [14], whereas elevated levels of vitamin D may result in the development of nephrocalcinosis and hypercalciuria [15]. These outcomes demonstrate the “U” shaped curve of nutritional status, with the risk increasing in deficient or excess states.

In addition to the tailored need for macronutrients, the micronutrient requirements immediately at birth and over the course of physiological development currently have the potential to be more closely evaluated. First, many physiologic changes that occur throughout hospitalization in the NICU may alter how these nutrients are metabolized, utilized, and stored within the body. For example, fat-soluble micronutrients (e.g., vitamins A/E/D and carotenoids) that are not circulating in the bloodstream can be stored in adipose tissue. This concept must be considered, as the percentage of body fat in a 1000 g infant changes dramatically from approximately 2% at birth [16] to roughly 16% at the term-corrected age and 24% at 52 weeks gestational age [17]. Second, the development of comorbidities, inflammatory and oxidative exposures, and the use of medication while in the NICU may further alter metabolism and the use of micronutrients [4]. Third, the effectiveness of past research on micronutrient supplementation (e.g., vitamin A) must be considered in the dual context of modern medicine and the effectiveness within younger surviving gestational ages. For example, Tyson et al., who provided intramuscular vitamin A in their evaluation to lessen the risk for developing bronchopulmonary dysplasia, reported initial results that were published more than two decades ago, and the average birth demographics of the subjects were larger than a micropremie and older than 25 weeks at 770 g and 26.8 weeks gestation [18]. While studies continue to evaluate the optimal vitamin A dose (per dose vs. dose/kilogram) and route (enteral vs. intramuscular) for administration [7], the effect of accumulative doses must be evaluated in a more significantly underdeveloped population. Accumulative doses must account for the amount received through supplementation, but also from parenteral nutrition or enteral feeding—in addition to the baseline nutrient status, which can vary widely at delivery [19]. Considering parenteral nutrition, ELBW infants receive a lower daily parenteral multivitamin dosing (1.5 milliliters daily = 690 IU/day) compared to those weighing >1000 g (3.25 milliliters daily = 1495 IU/day) [20]. Finally, the statuses of non-essential nutrients must be evaluated, such as those of carotenoids lutein and zeaxanthin, which are found in fetal tissue as early as 20 weeks gestation [21]. Past research in preterm infants indicates a trend toward favorable clinical outcomes when provided via enteral supplementation during NICU hospitalization [22]. However, continued research is needed to determine the full physiological effects during fetal development according to the method of delivery, the timing of delivery, and the quantity of intake.

## 3. Current Evidence

*Why do we still have nutrition-related questions about this smallest preterm population?* Many quandaries exist regarding the ideal theoretical versus feasible goals in providing nutritional care to micropremie and <25 weeks gestation infants, particularly in the first week/s of life. For example, the research on ELBW infants indicates that a higher energy provision may reduce the risk of developing comorbidities such as bronchopulmonary dysplasia and retinopathy of prematurity [23,24,25]. Yet in clinical practice, the smallest and most preterm infants may exhibit hyperglycemia with an increased parenteral glucose infusion due to stress from extrauterine adaptation, a decreased insulin release, and an increased insulin resistance [4]. While an increased energy provision may decrease the risk of developing certain comorbidities [23,24,25], hyperglycemia may increase the risk of inflammation, mortality, and the development of alternative morbidities such as worsened neurodevelopment and late-onset sepsis [4]. Hence, more research is needed to determine whether early alterations in nutrition metabolism result from lower initial energy needs during an infants’ adaptation to extrauterine life, or whether an extrauterine stress response simply causes the initial impaired nutrient utilization despite the high nutritional needs.

We have yet to determine the most optimal management strategies for enteral and parenteral nutrition with respect to the timing of initiation, the supplemental dosing quantity, the substrate used, and the method of advancement due to the variability or lack of evidence-based research. As previously acknowledged, the research on the extremely preterm or ELBW population is less concrete compared to larger infants, such as those born VLBW (<1500 g) or even at low birth weights (<2500 g). This is likely due to the smaller number of ELBW infant births, the inclusion of growth-restricted infants who are ELBW but not extremely preterm, the general variability in acuity after birth, and the increased challenges of conducting clinical research [4,26,27]. While quandaries may also exist in older or larger populations, these are less helpful in a more extreme population and when results may not be fully extrapolated.

The variability of the available literature is exemplified in regard to enteral feeding management. For instance, in VLBW infants (which also includes subsets of ELBW infants), a 2022 Cochrane review revealed that delaying the introduction of enteral feedings has no impact on the risk of developing necrotizing enterocolitis [27]. Likewise, the delayed introduction of enteral feedings results in intestinal villous atrophy [28,29] and prolongs the duration to achieve full enteral feeding [27]. Similarly, a 2021 Cochrane review concluded that smaller advancements of enteral feeding volumes compared with higher volumes do not reduce the risk of necrotizing enterocolitis or death in VLBW infants [26]. Yet, these data starkly contrast with the available published research on micropremies (<750 g), with the methods indicating that a lack of enteral feeding for up to the first two weeks of life followed by methodically small volume advancements decreases the incidence of necrotizing enterocolitis and the associated mortality [30]. However, these data must still be weighed against alternative research that reports a higher risk for developing bronchopulmonary dysplasia, retinopathy of prematurity, and comorbidities in infants born <33 weeks gestation who started enteral feedings after three days of age compared to before [31]. Given the sparse or differing results in the literature for varying preterm populations, we need more published data on the cohort of micropremie and <25 weeks gestation infants. Additional information may help in developing more standardized nutrition protocols within this subcategory of infants, with the importance summarized by Koletzko et al.: “Implementation of standardized feeding protocols in the NICU is a simple and inexpensive intervention that has resulted in improved outcomes…” [4].

## 4. Future Research

*What are some areas of future research within this specialized population?* As suggested previously, more research is needed to concretely determine the optimal strategies for detailed enteral and parenteral nutritional support. This includes research on the overall macronutrient needs at varying ages and stages of development, the timing of nutrition introduction, the nutrition source or substrate, and the method of nutritional advancement or modification. Management strategies should also analyze the immediate biochemical, physical, clinical, and developmental outcomes as well as the prospective and life-long results.

Likewise, future research can evaluate the detailed management of micronutrient needs. In addition to the extreme lack of intrauterine micronutrient accrual by the time of birth, extrauterine micronutrient needs may be altered in micropremies or those born <25 weeks gestation to account for differences in metabolism or nutrient utilization resulting from inflammatory exposures induced by medically necessary interventions [32]. The prolonged presence of inflammation is associated with the development of chronic disease [33]; consequently, modifications to the current standard nutritional interventions may have the potential to mitigate the negative consequences of inflammatory exposures. However, evidence-based research is needed to answer these questions and hypotheses more adequately.

Furthermore, the interconnections between essential organ systems must be considered in the comprehensive care of these vulnerable infants. For example, consider that the delayed start of enteral feedings may contribute to the occurrence of inflammation [31] and alterations to the intestinal microbiome [34]. Systemic inflammation increases the levels of pro-inflammatory compounds circulating in the blood, which may cross the infant blood–brain barrier [35]. An increased inflammatory exposure of the brain during critical periods is associated with worsened developmental outcomes, including increased mental health disorders, autism, and developmental delays [36,37,38]. Likewise, alterations in the intestinal microbiome are suspected to contribute to worsened developmental and health outcomes [39,40] given associations with other organs including the “brain-gut” connection and the even more novel “gut-lung axis” [41]. Thus, clinical questions regarding the enteral timing, quantity, advancement methods, and substrate are necessary to decrease the risk of adverse outcomes.

Additional topics of research may be novel and expansive. For example, amniotic fluid contains a variety of nutritive and non-nutritive components that benefit the development of the fetus, both internally and externally [42]. Considering the physiological stages of development, the continued evaluation of amniotic components and their effects on underdeveloped neonates may guide future nutritional strategies that promote the development or protection of critical organs when infants are born at periviable gestational ages. The evaluation of the related study outcomes may be immediate and/or long-range. The immediate outcomes may evaluate biochemical indices, inflammatory markers, serum amino acid or lipid concentrations, urine organic acids, or even epigenetic markers to evaluate the state of stress vs. homeostasis during the neonatal period. These initial outcomes will allow for a more detailed evaluation of early nutrition interventions, such as the provision of parenteral nutrition or the early introduction of enteral feeding. The intermediate outcomes will evaluate those throughout the NICU stay, such as mortality, growth, and the development of comorbidities such as BPD. More prospective outcomes will evaluate the neurodevelopmental outcomes and school performance, with the most long-range being health outcomes in adulthood. Studies evaluating the effect of specific nutrition interventions at these varying time points are critical. The evaluations at each timepoint provide useful information, but the collective and prospective culmination of these varying outcomes will facilitate the most comprehensive understanding to enhance the nutritional care of these high-risk infants. These outcomes will encompass decades of evaluation, but immediate action is necessary as we consider how current clinical practices have been influenced by past decades of research.

Future research may comprise retrospective, prospective, or cross-sectional results. It may encompass basic science, translational, or clinical research. Randomized-controlled trials are the gold-standard, but other methods of research may be needed in populations that may take time to acquire consented eligible subjects and acknowledge that survival rates will vary within a population at such high risk of early hospital mortality. Randomization at the unit or hospital level may be necessary to acquire information about the impact of important nutrition interventions as early as in the first hours of life.

## 5. Conclusions

The comprehensive and long-term nutritional care of the most extremely preterm infants must be closely provided, monitored, and modified as indicated. Infants born as early as 21–22 weeks will receive synthetic “intrauterine” nutrition for nearly half of the period of fetal growth and development. The insufficient or overprovision of nutrients during these critical stages is not benign and nutritional needs may change based on infants’ physiological development, the necessary medical interventions, the infants’ physical growth, and their body composition and size—all of which are changing rapidly throughout NICU hospitalization.

Given the fragility of micropremie infants and the current nutritional quandaries, research on the details and intricacies of the management and outcomes of medicalnutrition therapy must be continually evaluated and published. Only with reproducible and transparent methods, multicenter trials, and the sharing of data can we determine the most feasible and superior methods of nutritional care. While many variables surround delivery and the baseline demographic factors cannot be altered, nutritional care remains modifiable and past research has elucidated its role of critical importance.
